# “They Grow up in That Environment”: Latinx Immigrant Mothers' Sociopolitical Engagement, Youth Agency, and the Role of Community Organizations in a Divisive US Immigration Landscape

**DOI:** 10.1002/jcop.70123

**Published:** 2026-07-17

**Authors:** Fernanda Lima Cross, Saraí Blanco Martinez, Joel Lucio, Jasmin Aramburu, Gabriela Livas

**Affiliations:** ^1^ University of Michigan Ann Arbor Michigan USA; ^2^ University of Texas at Austin Austin Texas USA

**Keywords:** civic engagement, Latinx activism, Latinx immigrant, Latinx parent, Latinx youth, socialization, youth empowerment

## Abstract

Drawing from the healing ethno‐racial trauma framework we explore how Latinx mothers' understanding of sociopolitical issues and civic engagement potentially facilitates youth's own understanding and healing from the ethnic‐racial trauma experienced by their community. In‐depth interviews were conducted with six Latinx immigrant mothers and adolescents (12 individuals) involved in Cosecha, a grassroots immigrant rights organization. Thematic analysis resulted in two themes from interviews with mothers: (1) Familial activism and community organizing; and (2) Sociopolitical climate and socialization. Two additional themes emerged from adolescents' interviews: (1) Familial activism, fears and community support; and (2) Sociopolitical communication and awareness. Results suggest that mothers' participation in Cosecha promotes agency, empowerment, and healing among youth. Engagement in grassroots organizing may support healing and empowerment for mothers and youth. By engaging in efforts that affirm the human rights of immigrants, mothers model positive coping mechanisms. In turn, youth develop a greater sense of personal agency and belonging, and deeper knowledge of social justice issues in their communities through protesting.

## 1 Introduction

Immigration policy, and the treatment of immigrants in the United States, is a perennially divisive issue that has garnered significant national attention in recent years. President Trump's notorious anti‐immigrant platform continues to influence the current sociopolitical climate as we see political figures within the Republican party enact harsh legislation at various levels of government, especially as migrants increasingly seek help at the southern border (Romo 2023). Aside from legislation, we also see political tactics from various governors to bus migrants from their respective states and leave them in cities deemed immigrant‐friendly based on an alleged need for security and protection (Levin 2023). In response, we have been observing more displays of pro‐immigrant protests aiming to impact the rights as well as societal perceptions of immigrants. While immigrant rights groups have existed prior to the Trump administration and have secured enormous wins, such as the creation of the Deferred Action for Childhood Arrivals (DACA) program in 2012, the explicit anti‐immigrant rhetoric and restrictionism in national politics have escalated sociopolitical and civic engagement efforts among immigrant communities across the country, particularly within the Latinx community.

With the absence of national immigration reform and the exceedingly difficult challenges encountered at the federal level, much of the organizing among immigrant rights groups occurs at the state or local levels. Responses to xenophobic attitudes and policies have ranged from advocating for more sanctuary city policies to assembling rapid‐response teams against deportation threats and enforcement, with the latter often made up of volunteers and community members (Kocher 2017; Lopez et al. 2020). As we look toward Michigan, we see that this state is a microcosm for immigrant rights struggles for inclusion and equality. In fact, its residents continue to fight for policies that will help them meet their basic needs, with particular attention to identification issuance as many other states have also banned unauthorized immigrants from obtaining state‐issued IDs. For example, in 2007, Michigan's Attorney General issued an opinion that reversed a 1995 ruling that stated no section of the law prohibited unauthorized people from accessing a driver's license. Further action redefined guidelines requiring Michigan residents to prove legal US presence to obtain licenses or identification cards; this went into effect in 2008. Lack of a government‐issued ID poses several barriers to basic necessities: people are unable to access community resources or institutions such as health and human services, housing, employment, banks, and food pantries. Not having an ID can even cause problems when picking up children from school (Movement Advancement Project 2022). The risk associated with driving without a driver's license brings about particular challenges for unauthorized residents as traffic stops can be the onset for deportation procedures, especially when there is active collaboration between local law enforcement and US Immigration Customs and Enforcement (ICE) (American Civil Liberties Union 2022). Aside from the everyday disadvantages that unauthorized immigrants face when lacking proper identification or driver's licenses, the exclusion by design can be psychologically and physically draining, leading individuals to feel degraded, targeted, and ultimately isolated (ENGAGE 2023). Given these challenges, obtaining government‐issued IDs has become a critical focus of state‐level advocacy by immigrant rights groups.

In truth, the impact of exclusionary policies and practices such as limiting access to government IDs and licenses for unauthorized individuals extends to US‐born children and adolescents in mixed‐status families, or families whose members have varying immigration and citizenship statuses. In 2021, 35% of US‐born Latinx youth in Michigan had at least one immigrant parent (The Urban Institute Children of Immigrants Data Tool 2023). With the increase in open attacks on the Latinx immigrant community and thus the rising visibility of immigrant rights organizing (and organizing in general for other current social justice issues), parents are compelled to discuss the sociopolitical climate with their children (Blanco Martinez et al. 2023).

As these policies have implications for Latinx immigrant families, more broadly, we must then understand what factors lead Latinx immigrant parents to become publicly involved in their communities and how this involvement then impacts adolescents' sociopolitical engagement. Although more research is needed on the topic, involvement in an immigrants' rights organization may serve as a protective factor for immigrants as they navigate a hostile sociopolitical climate (Bañales et al. 2020). In this study, we engage with members of Movimiento Cosecha, a national movement with state‐level organizing that advocates for protection and respect for undocumented immigrants by working to reinstate access to driver's licenses for unauthorized immigrants in Michigan. Through interviews with mothers and teens, we explore how agency development helps mothers cope as Latinx immigrants in the United States, and in turn how involvement in community organizing serves as a tool to engage with their adolescent children and to support their civic agency as they also witness and journey through the sociopolitical climate.

We draw on the Healing Ethno and Racial Trauma (HEART) framework to guide the current study (Chavez‐Dueñas et al. 2019). The HEART framework, rooted in Liberation Psychology and trauma‐informed care, theorizes the healing potential of Latinx immigrant communities, while being cognizant of the effects of interlocking systems of oppression (i.e., racism, ethnocentrism, heterosexism, etc.). HEART draws from Indigenous and African beliefs stating that healing must attend to the emotional needs of individuals and the external oppressors in a cycle of four phases: (1) the establishment of sanctuary spaces; (2) acknowledging and coping with symptoms of ethno‐racial trauma; (3) connecting with community and healing practices rooted in ancestral cultural practices; and (4) liberation and resistance. Thus, we posit that community organizations can serve as spaces in which Latinx immigrant community members can feel safe from the daily persecution by immigration enforcement agencies. Community organizations can also be consciousness‐raising spaces in which people learn about the socio‐historical factors that contribute to their own persecution and that of other marginalized communities, as well as the histories of resistance and ancestral strengths that have supported the resilience of communities of color. In learning about shared struggles, community organizations can serve as spaces that facilitate cross‐cultural bonds and coalition building among diverse marginalized groups to challenge societal institutions that contribute to unjust social conditions. These factors have the potential to support the healing of Latinx immigrant community members as they are empowered to join in collective efforts to improve their communities and other historically marginalized people.

### 1.1 Political and Ethnic‐Racial Socialization

Youth sociopolitical development evolves across several contextual factors that include the current political climate which, in the United States, is inextricably tied to race (Robinson 2020; Bañales et al. 2021). For Latinx mixed‐status immigrant families, the hostile sociopolitical environment has led to greater necessity and urgency to discuss discrimination, documentation status, civic participation, and safety (Blanco Martinez et al. 2023; Cross et al. 2021). These discussions are regarded as ethnic‐racial and political socialization. Ethnic‐racial socialization (ERS) is the process by which parents share messages to their children and adolescents about what it means to be a member of their racial or ethnic group, including cultural traditions, how to navigate discrimination, and how to interact with others in a racialized society (Huguley et al. 2019; Umaña‐Taylor and Hill 2020). Importantly, mothers are commonly seen as the primary parent influencing youth's ethnic‐racial identity formation within ERS literature (A. T. Anderson et al. 2015; Kulish et al. 2019). In fact, mothers' favorable view of their own membership in their ethnic‐racial group and positivity toward others in said group has been linked to their children's own regard of their ethnic‐racial group through socialization practices (Kulish et al. 2019). An emerging subset of ERS is documentation status; that is, messages about what it means to be an immigrant, which often includes the potential dangers associated with unauthorized status (Cross 2022). Political socialization on the other hand, pertains to the process by which youth acquire political knowledge, beliefs, attitudes, and behaviors from their parents (McIntosh et al. 2007). These two processes tend to be considered as separate; however, they may overlap across politics and race as they are inherently intertwined (Bañales et al. 2021).

Not only have interpersonal instances of racism and anti‐immigrant sentiment increased in the United States, so has anti‐immigrant legislation and the policing of immigrant communities. This particularly affects darker skinned immigrants relative to their lighter skin counterparts as immigration has increasingly been racialized. For instance, law enforcement officers have been instructed to engage in racial profiling specifically by targeting people with “Mexican ancestry” in traffic stops, further imposing risk for Latinx immigrants as more police departments collaborate with immigration authorities (Fitzgerald et al. 2019). Additionally, conspiracy theories pushed by law makers and the media, primarily Fox News, such as the “great replacement theory,” have been used as a justification for the implementation of punitive racially motivated immigration laws (Gabbatt 2022; Rose 2022). The great replacement theory is a conspiracy theory endorsed by white supremacists in the United States to rebrand “white genocide,” or the idea that white people are being replaced with immigrants and people of color (Farivar 2022). Such xenophobic ideation has sparked several racially motivated mass shootings, including in El Paso, Texas where the shooter wrote a manifesto insisting on a “Hispanic invasion” followed by killing 23 people and injuring over 20 others, most of whom were of Latinx descent (Pskowski 2022; Abutaleb 2019).

As such, Latinx parents use both ERS and political socialization as means to increase youth's awareness of structural factors contributing to unjust policies (i.e., critical reflection) to navigate racism and xenophobia, and to maintain family safety (Blanco Martinez et al. 2023). This is in large part due to Latinxs, particularly Latinx immigrants, experiencing a politicized ethnic‐racial identity, making conversations about race, ethnicity, and culture a political discussion as well. Given adolescents' increased cognitive abilities and growing awareness of social inequalities, parents engage in greater levels of ethnic‐racial and political socialization during the teenage years (Pinetta et al. 2020). Previous research suggests that communication and emotional support received through ERS results in greater racial coping self‐efficacy, or “the belief that one can successfully challenge a stressful racial encounter,” among Black youth (Anyiwo et al. 2022; R. E. Anderson and Stevenson 2019). Preliminary findings suggest that parallel processes among Black and Latinx youth regarding ethnic‐racial and political socialization appear to occur, including how coping may cultivate critical reflection and sociopolitical action (Salcido and Stein 2023; Bañales et al. 2021; Pinetta et al. 2020). Critical reflection taught by parents can involve critical agency, the belief that an individual or community can enact change by challenging structures; which, in turn, results in sociopolitical action toward social justice. Indeed, in Latinx immigrant families, parental messages of preparation for bias and discrimination moderated the relation between immigrant policy related stress and critical action so that parental conversations potentiated action when youth reported greater immigrant policy stress (Rojas Perez et al. 2023).

Another way parents engage in political socialization is through civic modeling, which allows youth to witness political and civic socialization in practice, and which is exemplified by parents voting, participating in public demonstrations, or being involved in community justice‐oriented organizations (Bañales et al. 2021; Wray‐Lake 2019). ERS and political socialization, then, are not exclusively practiced by verbal communication. It also includes actions that parents take to transmit important messages about how youth should engage civically and politically (Huguley et al. 2019). Therefore, parent participation in community organizing spaces can be especially significant for the development of youth ethnic‐racial and civic identity, well‐being, and awareness of sociopolitical systems, power, and oppression. Thus, when parents engage in community organizing focused on increasing the rights of immigrant communities, they are socializing their youth to also value such work (Pinetta et al. 2020). In fact, even without engaging in community organizing, US‐born youth of immigrant parents with unauthorized status feel a general sense of duty to protect their parents and other community members in the face of racism and discrimination (Blanco Martinez et al. 2023; Wray‐Lake 2019).

### 1.2 The Role of Community Organizations

Community‐based organizations have historically provided important resources and services to individuals (Balcazar et al. 2009; Cicognani et al. 2015). Within Latinx immigrant communities, such organizations provide social support, education about sociopolitical issues, and opportunities for engaging in critical social action (Vasquez Guzman et al. 2020). Community organizations also help immigrant communities foster connections to their heritage cultures, creating a sense of community and belonging (Bañales and Rivas‐Drake 2022). For example, community members celebrate important cultural holidays and traditions (i.e., *Dia de los Muertos Posadas*). These practices provide opportunities for folks to engage in identity exploration, to learn about how their ethnic‐racial group is perceived by larger US societies, and to form conclusions about belonging to Latinx immigrant communities (Bañales et al. 2020). Identity exploration can also lead to learning about Latinx social positionality, different factors that contribute to the oppression and continued marginalization of their group, and ongoing efforts to resist and disrupt such systems (Mathews et al. 2020).

Furthermore, collaborations between community organizations and civically minded youth with strong connections to their ethnic‐racial groups have found creative ways to merge cultural practices with youth's civic participation. One notable example is Poder Quince/Quince Power by the Jolt Initiative launched in 2019. The Jolt Initiative is a Texas‐based nonprofit whose mission is to increase Latinx youth leadership and civic and political participation. Through their Poder Quince initiative, Latina youth use their *Quinceañeras* to increase voter registration by encouraging their party guests to register to vote. These efforts are a way to galvanize Texan Latinx communities into civic and political action as a direct response to voter suppression efforts in the state (Jolt Initiative n.d.; Flores 2019).

In addition to social support and access to resources, community organizing spaces can contribute to the mental and emotional well‐being of Latinx community members and youth. Youth who partake in community organizing are able to build resilience and cultivate radical hope that allows them to envision futures free of oppression and persecution, which incites them into action for social change. Thus, being involved in community organizing can be a healing experience for youth because they learn about the historical resilience and activism efforts that have allowed Latinx communities to survive in the United States despite ongoing, legally sanctioned persecution (Chavez‐Dueñas et al. 2019). Ortega‐Williams et al. (2020) found that youth of color who participated in community organizing efforts across New York were able to find a sense of belonging, protection, healing, and power in the collective. Youth found a sense of relief in their efforts and reported an empowering and therapeutic experience that contributed to their healing by sharing testimonies of their lived experience.

Movimiento Cosecha provides such a space for its members. Cosecha is a national immigrant‐led and nonviolent movement launched in 2015 with decentralized chapters in over 20 states. Their mission is to secure permanent protection, dignity and respect for the unauthorized community in the United States. Through noncooperation, organizers try to mobilize “the power of immigrant labor and consumption” to force a shift in power and public opinion, while simultaneously supporting diverse communities and their specific needs. Moreover, Cosecha tries to build solidarity across groups as their organizing tactics and strategies are influenced by other social justice movements. Cosecha focuses on immigrant protection and policy changes, including access to driver's licenses, know‐your‐rights workshops, and information on pathways to citizenship. Families that participate in Movimiento Cosecha often bring their children and adolescents, who can witness and even participate in dialogue and sociopolitical action centered on immigrant issues, including labor rights, racism, xenophobia, and detention (Cosecha Michigan 2023). For example, Cosecha responded to Florida's SB1718 by organizing, boycotting, and refusing to work across states. This law not only made it a felony to drive an unauthorized person, but also increased surveillance of legal status with employers, required hospitals to ask about documentation status, and invalidated driver's licenses from out of state for people with unauthorized status (Kenning 2023). Volunteers posted video calls to action on social media. Youth participated in these responses to raise awareness and provide education on SB1718's harm to communities (Cosecha Michigan 2023).

### 1.3 Current Study

The current study seeks to explore how Latinx immigrant parental involvement in an immigrant rights community organization, such as Movimiento Cosecha, has implications for familial well‐being with distinct experiences for parents and their adolescents. Specifically, we explore how mothers' involvement influences their ethnic‐racial and political socialization practices and impacts on youth mental health and civic engagement.

#### 1.3.1 Positionality

In this section, members of the data analysis team share their reflections on their positionality and reflect on the ways in which their varied social positions and life experiences may shape how they receive and hold the stories shared by the participants during the interviews, as well as how they approach the data analysis process.

I (Saraí) am a first‐generation Latinx immigrant who grew up in North Carolina in a mixed‐status family. I speak English and Spanish, and was raised in a household where Spanish was the primary language spoken. I have been a long‐time advocate of immigrant rights within my home community in North Carolina. Given my immigrant and advocacy background, I balanced my perspective as an insider as an immigrant rights advocate as well as an outsider as a researcher who is not from this region of the United States. Throughout data analysis, thoughtful conversations with co‐authors allowed me to acknowledge and confront biases.

I (Joel) am a 3rd‐generation Mexican American queer cisman from border town Laredo, Texas. I am able‐bodied, speak English and Spanish, and have birthright citizenship in the United States. I was raised in a family who all had birthright citizenship, but due to the proximity to the Mexican border, there remained a strong cultural and linguistic connection. Consequently, I related to the data and manuscript writing with “one foot in and one foot out” as an insider and outsider due to no personal experience of being in a mixed‐status family, yet with significant cultural and linguistic similarities. I also work as a Bilingual therapist with experience working with Spanish‐speaking immigrants, which provided greater in‐depth knowledge of the complexities this community faces as well as how citizenship status impacts mental health. Any preconceived assumptions were independently acknowledged and discussed with coding team members to reduce bias in data interpretation.

(Jasmin's) position as a second‐generation immigrant from a Mexican family has always influenced my desire to incorporate giving back to Latinx communities through my career choices. As such, I have experience providing social services to Spanish‐speaking communities. While I am a part of a mixed‐status family, no one in my immediate family has unauthorized status. Notably, my membership in Cosecha prior to the study may have influenced aspects of the research process: (1) feeling at ease interviewing the participants in my native language yet self‐conscious having to re‐introduce myself as an academic researcher rather than a member, (2) being able to understand the mechanisms through which the organization operates, and (3) my ability to relate to the social and political viewpoints expressed. While there was a familiarity with the community studied, I am not from the geographical location.

I (Fernanda) am a Latinx immigrant who was also a member of Cosecha prior to and at the time of data collection. I was involved in recruitment, conducting interviews in both Spanish and English, interacting with other members of Cosecha, data analysis, and manuscript preparation. As an immigrant and mother of adolescents, relating with participants who were also Latinx immigrants with adolescent children felt natural. Since I had engaged with Cosecha for almost 1 year prior to data collection, participants saw me as a fellow member rather than an outsider, which facilitated rapport as well as data analysis and write up. On the other hand, I have never been undocumented and thus was considered an outsider in that regard. Weekly team meetings were crucial for reducing biases and supporting analytical rigor.

## 2 Methods

### 2.1 Movimiento Cosecha Partnership

Given Movimiento Cosecha's important work, the PI aimed to establish a reciprocal relationship with them. She attended and was an active member of Movimiento Cosecha for a year prior to the data collection. Her participation included attending local chapter meetings and supporting local efforts and demonstrations. After integrating herself in the community, she offered to leverage university resources to support their cause. The other author who was more closely involved with Cosecha was part of the organization prior to joining the research team. Her participation in the organization was a result of her own interest in being involved more intimately with the community, specifically seeking to be a support system for immigrant groups in the area. The other authors did not have a relationship with Cosecha prior to the study. The PI approached Cosecha leadership and shared that her research interests focused on supporting the wellbeing of Latinx immigrant unauthorized and mixed status families. Movimiento Cosecha was interested in the partnership since the research could provide important data about their work which then could be used for grant applications to further drive their efforts. Cosecha's leadership enthusiastically took part in reviewing interview protocols and included questions that would benefit their movement. As such, they were also active supporters of our interview recruitment efforts. Once the interviews were conducted and the data had been analyzed, the research team attempted to get on their local meeting agenda to share the results with the organizations. However, by then, the leadership team had changed and the focus of the organization at the time was directed at preparing for May Day events and demonstrations that were happening locally and across the country. As such, the research team only shared the results with and received feedback from a few members of the leadership and was unable to present the findings for the entire team prior to publication.

### 2.2 Participants

The research described in this paper utilized data from a study that received approval from the Health and Behavioral Science Institutional Review Board (HSBS IRB) at the University of Michigan. Semi‐structured interviews were conducted with 12 Latinx immigrant mothers and their adolescents (6 adults and 6 teens) residing in Southeast Michigan. All of the mothers were immigrants from different Latin American countries (Mexico, Guatemala, Chile, etc.). Most of the adolescents were American citizens, but one of them had immigrated as a young child to the United States. This study did not intentionally choose to interview only mothers as we were interested in learning from the perspective of any parent involved with Cosecha. However, during recruitment, the only adults able to participate were women, despite interviews being conducted in the evenings or weekends. Interviews with mothers and adolescents were carried out separately via the Zoom platform between March and April 2021. Each interview lasted approximately 60 min and was recorded in audio format. Participants had the option to be interviewed in English or Spanish. All mothers opted to be interviewed in Spanish, while adolescents chose English. The interviews were conducted by four of the authors of this paper, who are all Latinx, bilingual, and bicultural.

### 2.3 Procedure

To recruit participating families, we collaborated with a local chapter of Movimiento Cosecha that, at the time, had about 15 members attending weekly virtual meetings. Cosecha helped disseminate digital flyers containing information about the study. The flyers expressed our interest in interviewing Latinx immigrant parents and their adolescent children aged 13–17 to better understand experiences within households, communities, and Cosecha. Adolescence is a formative period of sociopolitical socialization where the youth are developing a greater understanding of their ethnic‐racial backgrounds and awareness of the marginalization associated with being a member of a minority group (Pinetta et al. 2020). Thus, the age range of 13–17 was chosen given that youth in this age group are likely to still be living with their parents but also have enough experiences outside of the home leading to the formation of their own views and perceptions of the world separate from parents. Interested individuals reached out to the study team via text or phone. Upon contact, the eligibility of participants was assessed through a brief screening protocol conducted over the phone with the adult. During the screening, adults were asked about Latino/a/x identity for themselves and their children, age of children, and membership in Cosecha. Interested individuals were asked four eligibility questions: (1) Are you Latino/a?; (2) Are you a member of Cosecha?; (3) Is your child Latino/a?; and (4) How old is your child? If participants answered no to any of the questions or did not have adolescent children between the age of 13–17, they would be informed that they were not eligible to participate in the study. Participants who answered yes to all of the questions and had adolescent children between the ages of 13–17 were told they were eligible for the interview.

Several measures were implemented to protect the privacy and identity of the participants. Team members instructed the adults to choose pseudonyms for themselves and participating youth. These pseudonyms, along with the adults' phone numbers, were the only information saved from the participants. Once both the adult and adolescent were interviewed, their incentives for participation were sent via text message after which their phone numbers were deleted from our database and call or text history on Google Voice. Furthermore, a waiver of signature for the consent and assent forms was obtained from the IRB, and participants, including mothers on behalf of their children, indicated consent by orally confirming agreement to be interviewed and audio‐recorded.

Two semi‐structured interview protocols were created, focusing on mothers' immigration experience, communications between mother and child about immigration, racism, discrimination, and the broader sociopolitical climate in the United States. One protocol was tailored specifically for Latinx youth growing up in mixed‐status households, while the second protocol was tailored for immigrant mothers of mixed‐status homes. Mothers and adolescents were interviewed separately to better ensure the comfort of participants, and encourage them to express themselves freely. As such, participating mothers and youth only interacted with the research team member conducting the interview. Mothers were also asked about how their experiences with Cosecha impacted conversations with their adolescents. The adolescents were asked about their own perspectives and knowledge about the topics discussed by their mothers. Participants were free to turn their cameras on or off during the interview. Most of the adolescents chose to stay off camera while all adults kept their cameras on. Interviewers were intentional about creating a safe space throughout the interview by reminding the participants of the voluntary nature of their participation. The interview began by building rapport to help participants trust the interviewer feel more at ease while sharing important personal information such as current documentation status and parental socialization practices. The interviews were transcribed and translated by a professional company that adhered to a non‐disclosure agreement.

### 2.4 Data Management and Analysis

Our analysis team consisted of most of the authors of this paper, the principal investigator and three graduate students, who are all bilingual and bicultural. We read the transcripts, took detailed notes, and became well‐acquainted with the data. We managed and analyzed the English versions of the interview data files using the Rapid and Accelerated Data Reduction technique (RADaR; Watkins 2017) and reflexive thematic analysis. RADaR involved organizing each transcript into tables to facilitate revisions, team‐based discussion, and streamline the analysis process. RADaR takes place across four different phases that allow the research team to eliminate data unrelated to the research question and focus on condensed results. During the first two phases of RADaR, the research team worked in pairs to identify data from the interview transcript that did not directly relate to the topic of the study and removed it from the analysis files. The third RADaR phase involves the creation of a codebook in which the analysis team applies codes to each row of data that pertains to the topic. Subsequently, we developed a database of codes based on the team's notes and familiarity with the data. We assigned these codes and subcodes to the relevant data, drawing from the established database. During the fourth phase, the codes are used to create larger concepts that describe the interview transcript file. This process was repeated for each of the six adult interview transcript files as well as the six adolescent interview transcript files.

Next, we clustered the final codes and concepts into broader categories, which enabled us to identify overarching themes in the data (Bhattacharya 2017). Throughout this stage, our team met weekly to discuss the codebook, compare interpretations, and refine themes through collaborative reflexivity (Olmos‐Vega et al. 2023). Importantly, the HEART framework (Chavez‐Dueñas et al. 2019) informed these reflexive conversations during theme development and also guided our interpretation of the final themes. Specifically, HEART drew our focus to (1) how Cosecha functioned as a sanctuary space, (2) how mothers and youth acknowledged and coped with ethno‐racial trauma linked to immigration enforcement and racism, (3) how participants connected with community and engaged in healing practices rooted in shared histories, cultural strengths, and collective care, and (4) how civic engagement and organizing reflected liberation and resistance. In this way, HEART supported our analysis as we developed and named overarching themes and interpreted how socialization practices and civic engagement may function as mechanisms of healing that support youth's mental health.

Given our collaborative approach, RADaR made sense as our analysis method as it is an easy‐to‐learn and free data analysis tool as it can be done using Excel spreadsheets or Google sheet files. Furthermore, throughout the process, RADaR required team members to work individually then meet during each phase of the analysis process and reach agreement prior to moving on to the next phase, ensuring strong inter‐rater reliability. Team members documented areas of agreement and disagreement, resolved disagreements by referencing their notes, the codebook, or the original interview passages. In cases of disagreement, the team together tried to interpret participant responses accurately. Our team met weekly to discuss the codebook, emerging themes, and other details of the ongoing analysis. Additionally, we shared the results with our community partners, who provided valuable feedback that enhanced the study's validity (Grinnell and Unrau 2010). This meticulous process exemplifies the collaborative reflexivity of our analysis given that each phase of analysis included weekly discussion, in pairs first, and then and as a large group where decisions were discussed and challenged amicably, when necessary, thus ensuring rigor in the analysis (Olmos‐Vega et al. 2023).

## 3 Results

Two main themes were uncovered from mother interviews: (1) Familial activism and community organizing, and (2) Sociopolitical climate and socialization. In addition, our analysis indicated two main themes from adolescent interviews: (1) Familial activism, fear, and community support, and (2) Sociopolitical communication and awareness. We discuss each of these themes in detail below. In presenting these themes, we also draw on the HEART framework (Chavez‐Dueñas et al. 2019) as an interpretive lens. Across mother and youth narratives, Cosecha functioned as a sanctuary space and a site where families named and coped with ethno‐racial trauma, strengthened community connection, and engaged in resistance and liberation through organizing and civic engagement. Thus, in this study, socialization practices and civic engagement are conceptualized as part of healing insofar as they build agency and grow individual's capacity to navigate and challenge systematic harm.

### 3.1 Mother Theme 1: Familial Activism and Community Organizing

Mothers shared the impact that participating in Cosecha has had on their families: building community, learning more about issues they face, developing organizing skills, having mutual support, feeling empowered to continue fighting for immigrant rights, and participating in activism and demonstrations. Lidia, for example, shared about telling her child: “…if you continue to fight with me hand in hand, walking step by step, together, I tell you, someday we will be able to travel to where you want to go, to visit the relatives that you don't know.” This quote shows how activism becomes a shared family project and a way parents frame collective struggle as the pathway to future mobility and family reunification.

Mothers also emphasized Cosecha's motto, “that the struggle is for dignity, and for respect, that we deserve respect.” They shared examples of the mutual support that members extend to one another, including the help received throughout the pandemic:

We try to look out for the needs of the community, especially in these times where people obviously do not have the support of the government, and it is difficult to get some help that could, for example, save us a little bit from the anguish that we were living, not having how to pay the rent, how to have food when we stopped working. So, it is a movement that is always thinking about the well‐being of the families.

Lidia

This highlights Cosecha as a source of mutual aid and community care, especially during crises, when families experience exclusion from formal support networks.

When asked about how their involvement in Cosecha impacted the types of messages they share with their adolescent children, mothers explained that Cosecha has trained and empowered them to organize and advocate for immigrant rights:

Now our language has changed, we used to say, “Poor him, he'll get deported.” And now it's like, “We have to do something because we are prepared.” It has changed the actions. That's what we have basically learned. We are creating unity among the community and we are trying to get them to lose those fears, those insecurities, to say, “Enough is enough.”

Sofia

Sofia's quote demonstrates that growing their organizing skills through their involvement in Cosecha has reshaped not only how mothers talk about deportation and injustice with their children, but how their responses to injustices have also shifted, thereby illustrating a marked shift from fear and resignation to efficacy and collective action.

Mothers also shared other ways in which Cosecha enables family communication and exposure to sociopolitical issues in ways that are appropriate for youth, thus emphasizing intentional and developmentally appropriate political education that helps youth understand family migration histories while reducing distress:

We as a movement had planned to have a workshop to explain to the children all of this migration of their parents, to have it only with the children so that we could explain it to them so that they could understand it, according to their age, so that they could see it, not as we see it, but in their own way so that it would not affect them.

Lidia

Lidia's quote further demonstrates community care as she indicates that the planning of the workshop was a collective effort to ensure age‐appropriate understanding of current sociopolitical events. Thus, not only are conversations being encouraged within family units, but also across the Cosecha collective.

Mothers also mentioned using high‐profile cases of police brutality as examples of the power of the community voicing concerns and fighting for change to help move toward social justice: “The biggest example I can think of right now is the George Floyd case. Knowing that raising my voice peacefully will slowly create a change” (Lupita). They also discussed the need for Cosecha members to be above any political party affiliation and emphasized the need to speak up and create a pro‐immigrant movement, while being critical of political promises:

I could say that Democrats are the good ones, but during Obama's administrations, many immigrants were deported, too. So, are they good or not? We got to this point in which we better only support our own cause instead of supporting any government. Still we have to raise our voices to demand our human rights, because we are doing no harm to anyone. Our only sin is to search for a better life for our families.

Lupita

Importantly, the sociopolitical education received through meetings and protests hosted and supported by Cosecha enabled mothers to connect their struggles as Latinx immigrants with those of other ethnic‐racial groups, which bolstered their ERS practices. A mother spoke about the power of organizing in shifting mindsets toward greater cross‐group solidarity:

Sometimes [people] come to Cosecha looking for a very specific thing, right? “Well, I want a license,” but then in the process of organizing and talking, and these educational workshops and all that, well, we are also opening our minds, right? We are also learning to stop being racist, because many times Latinos are racist with Black (people), right? To understand the different situations that people live, the different struggles.

Violeta

Violeta's quote is a clear example of how participation in Cosecha expanded members' perspectives beyond single‐issue goals toward broader anti‐racist consciousness and cross‐group solidarity. In fact, Violeta also says that the most useful piece of knowledge from Cosecha has been learning about slavery, “I also find that there are many similar things that can be taken from the history of the anti‐slavery struggle.” In turn, mothers communicate the importance of learning about and showing up for other ethnic‐racial groups to their adolescent children. Although her children no longer wanted to go to Black Lives Matter protests after having gone a few times, Violeta told her kids, “No, let's go, we have to make numbers, we have to support, we have to show solidarity” (Violeta). This demonstrates how mothers attempt to translate solidarity into action by encouraging youth to “show up” for other communities even when it is uncomfortable or tiring.

Participants also mentioned teaching their youth about this country's injustices and that even Latinxs, immigrants, children of immigrants, and unauthorized immigrants still have rights that should not be violated:

As a member of the movement, we know that we also help other people who are like us and that is why I am talking to my children. I am in this group because I am fighting for you to respect me, for you to see that if I am here, that I have rights. Your rights were violated by not receiving any help from the government, only because you are the children of who you are. That is why I am here in the struggle, and I will continue in the struggle, that is why I ask you to support me and to continue walking with me until we get what we want, which is the papers. This time they are going to support me to go to Washington and to fight and that they feel satisfied that I never stood idly by, and I fought, and I say if it is carried out and we get something or we don't get it, we have the satisfaction that we tried.

Lidia

Lidia's quote frames organizing as both self‐advocacy and parenting: mothers teach children about rights, model persistence, and invite youth into collective struggle as a source of pride and agency.

Mothers reflect on how sociopolitical issues and policy decisions made in the United States, such as the policy change from 15 years ago that banned unauthorized immigrants from accessing a Michigan driver's license, have led mothers and family members to organize, advocate, and resist: “We have been in basically all the marches that Cosecha has had, and we always go with the children too. They grow up in that environment” (Rosa). Rosa underscores activism as a family socialization tool, where children learn political participation as normal and expected through repeated exposure. In doing so, mothers hope that their fearless activism can serve as examples for their youth:

We went to Lansing, and I was shocked because, as an immigrant, we can't do certain things, or we think we can't do certain things. There were police and it was like all that energy that yes we can. That it doesn't matter our status and that we don't have to be hiding, we have to have strength, that was in Lansing and the other one was in Detroit on May 1, 2019. It was over a thousand people, it was like, “I'm here and here I'm going to continue.”

Sofia

Mothers discuss joining community organizations as a strategy to change the governmental, legal, and public perception of immigrant communities:

We don't want crumbs from the government, we want something that we deserve, as workers, as people because at the beginning you feel like people don't know who I am, and we live in the shadows. They say you have to go out, show that you exist, show that you are not going to give up until you get what you want.

Lidia

Lidia's quote beautifully captures an organizing stance that rejects minimal concessions and instead pushes for full recognition, rights, and visibility for immigrant communities. This theme aligns with HEART's Phase 1 (sanctuary) and Phase 4 (liberation and resistance). Mothers described Cosecha as a space of mutual support, dignity, and community care that reduced isolation and fear, while also emphasizing organizing, and demonstrations as ways of building power to challenge anti‐immigrant conditions. Mothers' descriptions of learning histories of injustice and building solidarity across groups also reflect HEART's Phase 3 (connecting with community and culturally rooted strengths).

### 3.2 Mother Theme 2: Sociopolitical Climate and Socialization

Mothers shared the different ways they discussed sociopolitical and ethnic‐racial topics with their youth, including the country's overall sociopolitical climate, its effects on families like their own, their hopes for the new administration—including possible amnesty for immigrants—and racism in politics. Mothers used examples to explain the overlap between ethnic‐racial, immigration, and political issues:

The past four years were very stressful for our families. We saw how fast and how easy racism spread throughout the country. It's very sad to watch how humans don't tolerate other humans. I can tell, from my own experience, that prior to Trump's government, we had a lot of hope on Hillary. They promised to give us amnesty under Hillary's government… We had the whole paperwork ready to apply for that. In the end, all of those dreams came to an end as Trump took the presidency. We had many friends who were arrested by police, and then deported during Trump's administration. It was very sad. So, imagine how stressful [it] is to go out to work without knowing for sure If I'm ever coming back.

Lupita

Lupita's quote connects national politics to everyday family fear by showing how changes in administrations, broken promises, and heightened racism translated into real risks, arrest, deportation, and constant uncertainty for immigrants and their loved ones.

Mothers recount describing to their youth the harmful practices against immigrant communities, including deportation and the detainment of unaccompanied children and youth. For example, Rosa reports telling her daughter “that there are prisons for children, that some of the children you have seen in Cosecha have been in those prisons, that they are persecuted, that they die in the deserts, that they pay a lot to get in here” (Rosa). This mother uses graphic examples to help her child understand that immigration is not just a debate, it involves punishment, danger, and loss that can happen to children and to people in their own community just like themselves.

Mothers think it's important to talk honestly with youth about politics and its impact on immigrants of all ages. For example, mothers recognized that interpersonal instances of racism or discrimination are manifestations of larger social issues and openly discussing these topics may help reduce instances of youth internalizing blame:

…not telling them and not explaining to them is like covering the sun with a finger. In other words, they are going to experience discrimination, they are going to experience racism, they are going to experience ugly comments that they hear on television, or at school, or on the street, or wherever they go. So, I really prefer that they know how racist this country is, how badly they treat immigrants, so that they also understand that it is not just their fault, or that they are responsible, but anything that happens to them is greater, it is beyond them.

Violeta

Violeta explains that naming racism directly prepares youth for what they may face and helps them place harmful experiences in a broader social context of injustice, rather than blaming themselves. Similarly, mothers also remind youth that everyone is entitled to certain rights regardless of documentation status:

I always try to explain to them how, despite being an immigrant we have rights. But those rights are not enforced, so I try to talk to them and make them see a little bit of what immigration is. For example, what is happening right now at the border. As children alone are crossing.

Rosie

In her quote, Rosie also points to larger systemic powers that influence the immigrant experience by noting that while immigrants have rights, those rights are not always accessible to them.

Sociopolitical conversations between mothers and their teens often stemmed from either the mother or youth witnessing deportation of family or community members, conversations at school, and management of personal and youth emotional trauma:

I took my kids to their Spanish class and I remember we saw the ICE [Immigration and Customs Enforcement] cars but I never said anything to them because I didn't want them to get nervous because I know it affects them psychologically. But they are so smart that they realized when we got to the parking lot, they saw how ICE started to run around those workers. So they saw what can happen and the fear they felt when they saw that we were very close to ICE and how they ran around those people who were just working, they were not doing anything else to be detained.

Rosie

Rosie's quote exemplifies how children learn about immigration enforcement not only through adults' explanations but by witnessing it firsthand, and how mothers then must weigh protecting youth from anxiety while also helping them process what they saw.

While mothers shared political views and beliefs with their adolescents, they also believed that teens should independently form a political opinion based on information from other sources:

They learn it themselves. They say, “Have you heard what the president said? Did you see what the policeman did? Did you see what that person did?” Sometimes it's no longer necessary for us to explain to them because they already understand those things and they have their own opinions and say, “Why the president or why the government doesn't do anything if we also contribute?”

Lidia

Lidia highlights adolescents' growing political awareness, showing how youth actively interpret events and question fairness, especially when they see their families doing their part but not being protected. As such, mothers also report helping their teens understand the direct impacts that anti‐immigrant and racist legislation has on their lives; but they also consider their ability to understand political issues and acknowledge the drawbacks of sharing stressful information with them:

If a policeman stops me just for my features, he can stop me. Since I don't have a document he can take me under arrest, even call immigration. So our children have learned to grow more. We have had to teach them to grow more and to have a plan B with themselves. And it's fine they learn, but in another part, it's not so good because we don't let them grow up as children.

Lidia

Lidia captures how mothers prepare youth for racial profiling and possible family separation by teaching safety plans, but they also recognize that this kind of preparation forces kids to mature too quickly. This theme maps onto HEART's Phase 2 (acknowledging and coping with ethno‐racial trauma). Mothers explicitly named racism, anti‐immigrant policies, and immigration enforcement as sources of stress and fear, and they used sociopolitical and ERS to help youth understand that these harms are not their fault, which can reduce self‐blame while supporting preparedness. Mothers' emphasis on rights, “plan B” strategies, and developmentally appropriate conversations also illustrate coping efforts families use to navigate ongoing oppression.

### 3.3 Youth Theme 1: Familial Activism, Fears, and Community Empowerment

Youth in our sample demonstrated an awareness of the sociopolitical climate, including different movements for racial justice. Youth also described their families' active involvement in local community organizations such as Movimiento Cosecha and in protests, in which they participated alongside their parents:

It really did help me understand more about the rights or the laws considering the whole license thing that's going on. I guess in terms of my rights it helps me better understand how my freedom of speech comes in hand because I've never really used it as much as I've had in protests.

Cielo, 14

Cielo highlights how her participation in Cosecha empowered her, specifically by helping her gain a better understanding of the law and how she can meaningfully enact her freedom of speech through protests in ways she had not done before.

Youth discussed how they engaged through Cosecha in community organizing and demonstrations alongside their parents. They shared that though organizing with Cosecha was an empowering and educational experience, being designated speakers at protests was an emotionally charged and overwhelming experience:

The first time I participated it was a march in downtown. I'm pretty sure my mom talked about it with me and I was like, oh, that might be nice so I joined in with her. That was my first ever involvement in terms of being a part of a march with Cosecha. So then the second time, I think there was a hearing downtown…I talked there as well. It was actually really emotionally overwhelming for me because I hadn't really done that before, so I kind of cried in the middle of it.

Cielo, 14

This quote shows how family‐based activism draws youth in, but also how public speaking about immigration issues can be intense and overwhelming because it requires them to share personal fears and experiences in front of others.

Similar to feeling overwhelmed, youth also reflected on their initial fears when participating in protests as it exposed them to possible discrimination:

I felt a bit worried if anybody was going to discriminate against us or say random racist remarks while we were out there. But luckily that didn't happen. People that went by, it was like, “Woohoo, we support it.”

Alejandro, 13

Protesting can feel risky for immigrant and Latinx youth because they anticipate racist backlash, yet supportive reactions from bystanders can reduce fear and reinforce the feeling that the community is not alone. As such, despite initial apprehensions, youth explained that their participation in Cosecha helped them feel part of their community which helped them deal with fears about their families' status in the country:

You can stand up for your community, and when you're standing up, you don't have to be afraid of things, like people telling you stuff, and how you could try to defend your community or defend yourself sometimes.

Eva, 13

Eva's quote indicates that partaking in collective action can replace silence and fear with confidence, because organizing as a collective gives youth a sense of protection, language, and strategies to respond to discrimination.

Youth also shared their desire for a fairer, healthier, more just world, for an end to racism and for respect and dignity for the immigrant community, which they hoped to achieve through their actions: “I hope that immigrants are treated more fairly, are welcomed more than we are right now,” (Alexandra, 13) and “I just want it to be a more welcoming place in which people can feel safe in and not have to fear about problems that are going on right now like being hate crimed” (Eva, 13). Together, Alexandra and Eva describe a core goal of their activism, shifting from exclusion and hostility toward a more welcoming and fair society where immigrants are treated with dignity and people can feel safe, recognizing that racism is not only hurtful language but also a real threat of violence (e.g., hate crimes) that shapes everyday life. Thus, youth felt empowered to create change and spoke of their obligation to contribute towards the immigrant cause given the tools that Cosecha has provided them:

The biggest support they have provided is just giving us an outlet in which we can voice our concerns or our experiences. I have talked about my claim as to why immigrants should get licenses, it's just giving us a platform in which we can speak on.

Cielo, 14

Similarly, youth also felt that it was a collective responsibility to work toward healthier communities:

Everyone should try to create these kinds of changes… I think that if my community is healthy, I'll have a better life… My community should be healthy, and that would benefit me, and also—well, I should help just cause…it's the right thing to do.

Victor, 15

Together, Cielo and Victor describe Cosecha as empowering because it provides a concrete platform to voice their experiences and concerns while framing participation in change efforts as a shared responsibility, both for community well‐being and because it is part of their duty as community members. Consistent with HEART's Phase 1 (sanctuary) and Phase 4 (liberation and resistance), youth described protests and organizing as initially risky and emotionally overwhelming, yet made more manageable through participating alongside mothers, receiving encouragement from community members, and having an outlet to speak publicly. At the same time, their concerns about discrimination and family status point to ongoing ethno‐racial trauma that youth navigated through collective action.

### 3.4 Youth Theme 2: Sociopolitical Communication and Awareness

Youth reported being aware of racial injustices, marginalization, and discrimination toward the Latinx community. This awareness reflects personal experiences in society and socialization received from parents and through the media. Nevertheless, youth still struggled with making sense of the situation: “It's really hard to grasp that idea that you hate someone else just because of their ethnicity or their race or their skin color or even the language they speak” (Cielo, 14). Cielo clearly expresses confusion and disbelief at the irrationality of racism, showing that awareness does not erase the emotional difficulty of understanding why racism and discrimination happen. Furthermore, youth also reflected on their own experiences with racism and discrimination:

At restaurants, for example, the manager once heard us talking in Spanish and he basically tried to in that kind way, not really, kick us out because I guess we were somehow scaring the other customers or something. It made me feel hurt, but also, I don't know, a bit frustrated because I didn't understand how two adults, a preteen and a toddler could be considered, I don't know, dangerous.

Alexandra, 13

Alexandra's reflection exemplifies how everyday spaces can become sites of discrimination, where speaking Spanish is treated as a threat, leaving youth feeling both hurt and confused by the stereotype of Latinx families as “dangerous.”

Adolescents also identified Donald Trump's hateful rhetoric directed at Latinx immigrants as playing a role in the anti‐immigrant sentiment that has permeated this country:

If I were to pinpoint on someone who's shown discrimination towards my community [it] is probably Donald Trump. Many of the things he said are really hurtful. I guess he enforced that whole stereotypical idea about us that really isn't true. I was really hurt by that. I never really supported him but still me hearing that, it's hurtful considering he was president. He should be making his citizens feel safe in their own country, but he just instilled fear.

Eva, 13

In her quote, Eva connects harmful political rhetoric to community climate, arguing that when leaders normalize stereotypes and hostility, it legitimizes fear and makes immigrants afraid to navigate society.

While youth were able to recognize the harmful impacts of racist rhetoric and experiences of discrimination, Youth also reported feeling a sense of relief by engaging in conversations with family members about how the sociopolitical climate impacts their community:

I feel like it's really good. It helps you kind of relieve stress and finally be able to talk about it with someone, even if they have different views on certain parts but you still kind of agree for the most part, especially since I feel like it's much easier to talk to someone in the family about these things than someone else.

Cielo, 14

Cielo's quote indicates that these family conversations function as a source of emotional support, where they can find reprieve by sharing fears and opinions and reduce stress. She also indicates that having these conversations within the sanctuary of the family unit feels safer than talking with outsiders.

Furthermore, when engaging in conversations with parents, youth indicated that they were active participants rather than passive recipients, as they also played a role in sharing information with their parents and helping them navigate that information:

I do that with my parents all the time. Usually they're not really well‐informed of some things, or they'll just focus on one news outlet and won't look at others. So not them believing in misinformation is really important, or just hearing about other sides of the story is really important because not everything is true. You have to kind of look at other perspectives to fully understand the true story. Or they won't exactly hear about something, so I'll pass it on to them.

Eva, 13

Eva's quote exemplifies youth as active information brokers in the family. Eva describes how she helps parents avoid misinformation by encouraging multiple sources and sharing updates they may miss.

Additionally, youth displayed an active understanding of immigration‐related processes such as deportation and the role of ICE that was rooted in their personal experiences:

It all started with my uncle. He used to live with us. My mom told us, because my cousin, she was crying because my uncle was caught by ICE and was taken to jail. Yeah. It was hard for her because it's her brother.

Alejandro, 13

They also reflected on additional triggers that lead parents to discuss politics at home:

Sometimes it's from family members. I guess something will happen to one of our family members, and my parents will bring it up. Most of the time I'm really confused as to why that happens, and they explain it to me, which also includes the political stuff.

Alexandra, 13

Both Alejandro and Alexandra show that youths' understanding of ICE and deportation often comes from painful, immediate family experiences that trigger political conversations at home, where parents help them make sense of confusing events by explaining the broader policies and power dynamics involved.

When asked about being informed on current events, youth explained that “it can be devastating, hearing about what's happening and not being able to do that much or feeling like you can't really do that much to help” (Alexandra, 13). Nevertheless, they explained that “it's important to stay informed from a young age. Because we can be more alert about what's going on to start developing more of an activist mind” (Alexandra, 13).

Furthermore, the teens also shared insights on parental need to engage in sociopolitical and ERS:

They would sit me down and talk with me and tell me all these things, and I would just listen. They also felt more kind of relieved in terms of stress talking about this with me. It was really nice to just see their stress somewhat kind of lower.

Eva, 13

Alexandra and Eva illustrate that staying informed can feel devastating and helpless for youth who recognize injustice but have limited power to change it, at the same time, they view early awareness as important preparation for future action. They also describe mother–child sociopolitical conversations and information‐sharing as both educational and stress‐relieving, helping families make sense of painful and confusing events (e.g., ICE and deportation). Together, these accounts align with HEART's Phase 2 (acknowledging and coping with ethno‐racial trauma) and Phase 3 (connecting with community and shared knowledge/strengths).

## 4 Discussion

Our findings provide an overview of Latinx mothers' participation in a grassroots community organization and explored how their ethnic‐racial and immigration experiences within US society shapes youth civic development. We also examined youth's perspectives on socialization received as well as how mother‐ and self‐involvement in an immigrant rights organization supports civic agency. These insights underscore the critical role of sociopolitical and ERS, community organizing, and collective action in addressing the challenges faced by immigrant families. Such efforts to engage in collective activism as a family can foster a sense of agency, healing, and empowerment among youth.

To interpret these findings, we draw on the HEART framework (Chavez‐Dueñas et al. 2019), which conceptualizes healing as a liberation‐oriented process that includes building power to navigate and challenge systematic harm. HEART guided the research team's reflexive conversations when creating overarching themes and guided our interpretation of the data. Thus, sociopolitical and ERS and civic engagement are conceptualized as mechanisms through which healing can occur in and through community organizations. This study provides a unique contribution to the larger body of research by extending the HEART framework to Latinx immigrant families engaged in grassroots activism. Previous examinations of HEART focused on individual healing or clinical settings, while our findings show that active family participation in community organizing (such as through Movimiento Cosecha) can also facilitate healing. In examining mothers' and youths' perspectives, we demonstrate how healing and empowerment occur across generations within sanctuary spaces outside of the clinical context. The work of this study contributes novel insights on how community organizations and groups can serve as critical sites of ERS and civic development, expanding theoretic understanding of how Latinx immigrant families experience empowerment, resistance, and healing against systemic oppression.

Mothers involvement in Cosecha affected their socialization practices. Not only did mothers come to see systemic discrimination and racism from an immigration standpoint, but as an issue against communities of color. This fueled solidarity and action across ethnic‐racial groups and their struggles. Because mothers utilized critical reflection and civic modeling as part of their ERS, they facilitated critical agency, the belief that an individual or community can enact social change (Bañales et al. 2021; Anyiwo et al. 2022). As a result, mothers were able to foster a sense of advocacy through sociopolitical action among the younger generation. Thus, our findings extend the scope of the HEART framework by also pushing for youth oriented healing spaces within communities.

Mothers involved in Cosecha were identified as serving as central socializers, meaning they intentionally engaged their adolescents in critical conversations around identity‐specific topics of systemic discrimination, racism, and immigration policy. These conversations are noted to be grounded in mothers' own evolving conceptualization of oppression, which is used as a tool to prepare their children for the realities of the sociopolitical climate. Adolescents reflected that these dialogs with their mothers served as an entry point into developing critical awareness and a sense of importance for community engagement.

Still, it is important to note that though mothers and youth engaged in Cosecha together and were each able to grow their critical consciousness, the growth sometimes manifested differently. For example, through the familial involvement with Cosecha, both mothers and youth recognized how the sociopolitical climate impacted their lives. Similarly, both mothers and youth described how participating in Cosecha enhanced understanding of laws and increased their awareness of their rights, which allowed them to grow their feelings of empowerment. These findings align with the HEART framework's Phases 1 and 2, finding a sanctuary space and acknowledging and coping with entho‐racial trauma. Specifically, within the Cosecha sanctuary space, youth and mothers were able to find support in each other as well as in other community members. Thus, both mothers and youth expressed a sense of belonging and camaraderie within the organization, enabling them to form connections to others who also came from mixed‐status immigrant families.

Part of connecting with other members and participating in Cosecha also often involved learning about histories of injustices and resilience within Latinxs and other communities of color. This aligns with Phase 3 of the HEART framework: connecting with community and healing practices rooted in cultural traditions. However, one way in which their growth manifested differently was through the use of social media, whereas social media usage was not an issue brought by the mothers. Indeed, youth reflected on their roles in disseminating information about social issues through social media and their participation in protests—as informed by lessons learned from maternal socialization efforts. Many youth shared that their motivation to engage in community organizing was rooted in dialog and modeling from mothers, thus highlighting intergenerational transmission of advocacy within immigrant families. They saw these actions as crucial for advancing social justice and challenging discriminatory policies, which points to Phase 4 of the HEART framework: engaging in resistance and liberation. Another way in which both mothers and youth differed was their approaches to engaging in protesting. Mothers saw engaging in protests as crucial to their sociopolitical development whereas youth expressed feeling more hesitation and concerns regarding how they would be received by the general public. Despite initial concerns about potential discrimination during protests, the youth reported receiving support and encouragement from bystanders (Chavez‐Dueñas et al. 2019).

Still, it is important to note that although we identified examples of all four phases of healing identified in the HEART framework, healing did not necessarily take place in the order outlined by Chavez‐Dueñas et al. (2019). As such, we can confidently state that there is evidence for healing within the overarching data and themes presented, as well as evidence that not all participants began their healing journeys with Phase 1 of the HEART framework. Indeed, some members sought out Cosecha as a place of sanctuary in response to either witnessing or experiencing first hand effects of immigration enforcement tactics within their families and communities. Others began the healing process in their homes by having sociopolitical discussions about the environment, and were emboldened to join Cosecha as a result of these conversations taking place within the family setting. As such, both youth and parent participants were in different stages of their healing process, and also began their healing process within different phases of healing theorized by the HEART framework, depending on their individual levels of critical consciousness before entering the Cosecha sanctuary space.

Mothers also described the ways in which they engaged in discussions with their teens about the sociopolitical climate in the United States, including hostile policies that specifically target Latinxs. As a result, Latinx immigrants develop a politicized ethnic‐racial identity because racialized immigration and law enforcement policies, coupled with the racialization of Latinxs, then put mixed‐status families at additional risk of experiencing harm. Aligned with previous research, our findings indicate that families that engage in these dialogues are better prepared to handle instances of discrimination and racism (Cross et al. 2020; Eyal et al. 2023). For both mothers and youth, engaging in such conversations is often prompted by real‐life events affecting the whole family unit, such as experiences with deportation or discrimination (Blanco Martinez et al. 2023; Bishop and Medved 2020; Cross et al. 2020). Mothers felt a responsibility to educate youth about challenges faced by immigrant communities and about the need to advocate for their rights, emphasizing that sharing these realities is important to foster awareness and understanding. As such, adolescents demonstrated a strong awareness of racial injustices faced by the Latinx community. As a result, both mothers and youth highlighted the importance of engaging in open and honest discussions as a family unit about the sociopolitical climate to better foster understanding and solidarity. Despite their understanding of these issues, youth found it challenging to comprehend why individuals would discriminate against others based on ethnicity, race, or language. Thus, while mothers acknowledged the necessity of these conversations, they also expressed concern about the potential psychological impact on youth. Conversely, youth expressed the need to engage in these conversations to expand their understanding of such issues.

Mothers shared the positive impact of their involvement in Movimiento Cosecha, as expanding their familial and personal agency. Individually, mothers highlighted how participation in these groups allowed them to build a sense of community with other Latinx immigrant families, learn about relevant political issues, develop organizing skills, and provide mutual support to other immigrants. As a family unit, mothers felt empowered to continue fighting for immigrant rights alongside their adolescent children and recognized the importance of collective action, specifically, the importance of involving youth in activism focused on challenging oppressive systems. Cosecha was crucial in changing their language and actions, evidenced through the socialization practices they engage their youth in, to encourage them to advocate for their rights. Through their sociopolitical socialization practices and engaging in activism together, mothers taught their teens about their agency to transform their worlds into a better place. As such, youth internalized these messages and felt empowered to make a difference to improve the lives of their families. These findings follow the HEART framework in which mothers and youth found a sanctuary space (Cosecha), coped with ethno‐racial trauma (social support, processing via discussions), connected with community and healing practices rooted in culture, and engaged in liberation and resistance (protests and demonstrations) (Chavez‐Dueñas et al. 2019).

Our findings also expand current research focusing on the healing potential of being involved in community organizing and activism (Ginwright and Cammarota 2002). Indeed, Chavez‐Dueña et al. (2019) argue that individuals can experience radical healing from ethno‐racial trauma when they are able to critically reflect and identify systems of oppression, such as anti‐immigrant policies, that contribute to their continued marginalization. Not only that, but the healing happens within sanctuary spaces (Chavez‐Dueñas et al. 2019), in our case a community organization, in which community members feel free from persecution, are able to grow their awareness of interlocking systems, and build connections with other community members. Youth who do not have parental involvement in an immigrants rights organization often develop preventative and protective mechanisms against instances of discrimination, racism, or possible detainment and deportation (e.g., avoidance, confinement, personal responsibility; Blanco Martinez et al. 2023; Cross et al. 2021); while youth with parents involved in movements are more likely to be encouraged to speak out about interpersonal and systemic injustices and identify structural barriers such as racism, anti‐immigrant laws, and policies, as the root cause of these issues. In addition, through the connections made within sanctuary spaces, adults and youth alike are able to find community and mutual support, develop healthy coping strategies, grow their civic agency, and hope that engagement in social action will benefit their communities. Therefore, participating mothers and their adolescent children can experience the radical healing process through activism and organizing efforts as members of Cosecha.

### 4.1 Implications for Practice

Our research has important implications for practitioners, policymakers, and researchers providing services for or conducting work alongside immigrant or mixed‐status Latinx communities. Practitioners and policymakers can commit to not collaborate with ICE agencies allowing for sanctuary spaces that facilitate the radical healing process. Similarly, researchers can engage in community‐based and participatory‐action research that centers community members as experts in their own experiences and challenges traditional notions of researchers as being the creators and gatekeepers of knowledge. Lastly, by working closely with community organizations providers can ensure that their services are culturally relevant and sustaining, empower and provide educational opportunities, and meet the needs of Latinx immigrant communities in tangible and practical ways.

As expressed by participants, legislative changes at the federal and state levels are as necessary as interrupting racist and xenophobic actions at the interpersonal level. Anti‐immigrant policies directly contribute to material hardship experienced by mixed‐status families, where children and youth are excluded from economic safety nets to which they are entitled as US citizens. Moreover, parents may not seek help due to misinformation about becoming ineligible for permanent status (Acevedo‐Garcia et al. 2021). Policymakers must understand the interplay between documentation status and eligibility for services and benefits, and craft legislation that no longer ostracizes people who contribute to the social and economic well‐being of communities across the country.

Youth of color are emerging as civically engaged voters and leaders in an environment that pushes them to think about the intersection of their identities and about how perceptions on race, politics, and immigration shape their future (Cureton and Aguinaldo 2023). As Latinx youth become increasingly larger in number, professionals working alongside this group must create pathways that amplify their voices and allow them to drive the change needed so that their communities are no longer reactive to legislation but proactive in shaping it.

While grassroots community organizations, such as Cosecha, are supporting healing and sociopolitical change, practitioners must increase youth participation in these efforts by facilitating peer‐to‐peer collaboration, for example. This study largely focuses on vertical political socialization; that is, we primarily focus on parental (from mothers specifically), top‐down political messaging and modeling behaviors. However, youth also undergo political socialization from peers, otherwise known as horizontal political socialization (Terriquez et al. 2020). Peer relationships can serve as a catalyst to promote positive development and prosocial behaviors, and practitioners can facilitate such dynamics in peer groups across community organizations like Cosecha (Mushonga and Henneberger 2019). Youth interviewed for this study shared how they disseminated information, dispelled myths, and debunked misinformation across social media to help their communities (Patel et al. 2023). Though internet activism has its strengths, practitioners can assist youth in having more embodied experiences within their communities, facilitating greater agency and empowerment (Erlick 2018).

### 4.2 Limitations and Future Directions

Though our research has notable implications for research and practice, there are also limitations that need to be addressed. One important limitation is that we are unable to examine additional youth outcomes, such as academic and physical well‐being, as well as their overall civic engagement. As such, future research should focus on how participation in community organizing and activism impacts youth educational, civic, and physical well‐being. Another important limitation to note is that most of the participating adults were women; as such it would be important to also hear the perspectives of fathers as well as other adult caregivers. Although this study included participation from six mothers and six adolescents, analyses were conducted at the individual level rather than within mother–adolescent dyads. As a result, we were unable to directly examine how perspectives within each pair converged, diverged, or directly influenced one another. This limits our ability to draw conclusions about relational processes and bidirectional dynamics. Future research should employ dyadic analytic approaches that explicitly link caregiver and adolescent data to better capture these interpersonal dynamics and provide a more nuanced understanding of how family members shape one another's experiences. Furthermore, although we interviewed six total mother–youth pairs with varying degrees of involvement, only five of the pairs were highly involved members of Cosecha at the time. Future research should focus on recruiting additional participants of a more diverse range of members to gain a more nuanced understanding of the impacts of participating in an immigrant rights community organization. Lastly, given the restrictions of the COVID‐19 pandemic, interviews were conducted in a virtual setting, which can make it more difficult to establish rapport with the participants.

## 5 Conclusion

Latinx immigrant and mixed‐status families are living and developing within a hostile sociopolitical climate that politicizes racial‐ethnic identities, increasing the need for parents to discuss race, ethnicity, immigration status, and politics with their youth. Drawing from the HEART framework, this study examines the role of ethnic‐racial and political socialization, community organizations—Movimiento Cosecha, specifically—and sociopolitical action in the healing and empowerment of Latinx youth. We found that, indeed, community organizations should be considered promoters of healing as they supported both mothers' and youth's understanding of the sociopolitical climate, increased well‐being and a sense of community, and provided clearer and actionable steps for families.

## Author Contributions

**Fernanda Lima Cross:** conceptualization, formal analysis, writing – original draft – revision. **Saraí Blanco Martinez:** conceptualization, formal analysis, writing – original draft – revision. **Joel Lucio:** conceptualization, formal analysis, writing – original draft – revision. **Jasmin Aramburu:** conceptualization, formal analysis, writing – original draft – revision. **Gabriela Livas:** writing – original draft – revision. All authors have approved the final article.

## Ethics Statement

The study was approved by the Institutional Review Board on the University of Michigan's Health and Behavioral Science (HSBS) Institutional Review board approval HUM00189045 on February 25, 2021. All research procedures involving human participants were in accordance with the ethical standards of the institutional and/or national research committee and with the 1964 Helsinki Declaration and its later amendments or comparable ethical standards.

## Consent

Informed consent was obtained from all individual participants included in the study. The authors affirm that human research participants provided informed consent for publication of their data.

## Conflicts of Interest

The authors declare no conflicts of interest.

## Data Availability

The data that support the findings of this study are available on request from the corresponding author. The data are not publicly available due to privacy or ethical restrictions.
